# Use of Recycled Fine Aggregates from C&DW for Unbound Road Sub-Base

**DOI:** 10.3390/ma13132994

**Published:** 2020-07-05

**Authors:** Luc Courard, Mélanie Rondeux, Zengfeng Zhao, Frédéric Michel

**Affiliations:** Building Materials, Urban and Environmental Engineering, University of Liège, Allée de la découverte 9, 4000 Liège, Belgium; luc.courard@uliege.be (L.C.); m.rondeux@hotmail.com (M.R.); frederic.michel@uliege.be (F.M.)

**Keywords:** recycling, construction and demolition waste, recycled concrete aggregates, road sub-base, fine, shape, compaction

## Abstract

Fine recycled aggregates are produced in large quantities when crushing Construction and Demolition Waste (C&DW). Even if coarse recycled aggregates are commonly used for road foundations, fine particles are often rejected as they are considered detrimental for the long-term behaviour of foundations. Physicochemical, mineralogical and mechanical characterizations (through X-ray diffraction, X-ray fluorescence, the chloride and sulphate contents, Los Angeles abrasion, micro-Deval resistance and static plate load tests) were performed on raw and treated fine recycled materials for understanding both the effects of the preparation, the compaction and the freeze–thaw cycles on the properties and the evolution of fine particles. Special attention was provided to the shape analysis of fines by means of image analyser. The results showed that the main characteristic parameters to be considered are the sieving curve and the proportion of grades. The mixes containing the highest quantity of fine particles, specifically lower than 63 µm, usually inducing a higher water demand and a higher capillary rise. This can be explained by specific surface and bluntness parameters which increase with the finer particles, inducing a higher surface roughness and, consequently, a higher potential interaction with water. Compaction did not seem to have a major effect on the production of fines (despite some breakdown occurred during compaction) and on the shape of materials (the bluntness and convexity increased slightly, while the elongation values remained similar after the compaction process). The static plate load tests showed that bearing capacity is slightly lower than the specifications for the road foundation after compaction. However, the studied material could meet the maximum criteria for secondary roads foundation construction on the wear resistance criteria. Recycled aggregates from C&DW without sufficient quality could be blended with other aggregates to enable their usage for upper-level road foundation.

## 1. Introduction

The durability of an aggregate used in a road structure can be defined as the capacity of the material to be stored over time and to preserve its initial characteristics, in particular, its particle size distribution vs. various stresses (since its treatment until the destruction of the road) [1,2]. This definition involves three key concepts that should be specified, namely the conservation of the particle size distribution, the life cycle of material and the defects by solicitations present within the material [3,4,5].

An unbound granular mixture is an assembly of grains of different sizes and nature that touch each other at different points of the surface and are separated by intergranular spaces filled, with either free water or capillaries or air [6]. Each phase is characterized by distinct properties that influence the overall behaviour of the material [7].

In the presence of stresses within the material, a part of the stresses is concentrated in the liquid phase (in the form of interstitial pressure), whereas the other part is concentrated in the solid phase and, in particular, in certain zones such as the contact points between grains, areas of weakness of the rock (cracks, pores, etc.), or at the water–solid interface [8,9,10]. If these stresses grow, they can cause fractures within the grains as well as their polishing, which results in their decomposition and the formation of fine particles [11]. Therefore, this causes a change in the granular structure of the material that often results in the loss of geotechnical characteristics [9,12], not only of the layer concerned but also of the entire structure [13].

The conservation of the particle size distribution is therefore an important factor in the durability of an aggregate [14,15]. This is why the majority of sustainability tests provide results in the form of fines produced during these tests [16]. The presence of fines within a granular material can have several positive or negative effects depending on the content on the following three parameters [17,18,19,20,21]:

(a) The compactness, the bearing capacity of the material as well as the resistance to the different types of deformations: the fines tend to fill the intergranular spaces during the compacting operation [22,23]. This improves the density of the material and therefore its compactness. Therefore, from a certain content (between 20–25% depending on the initial porosity of the coarse material), the addition of fines generates a reverse phenomenon, namely a decrease in density [24]. The fines no longer occupy only the interstices but they separate the large elements and the initial porosity of the material becomes more and more important [25]. It should be noted that the specifications allow a fines content of up to 9% for load bearing layers such as the foundation.

(b) Permeability: the fines present in the interstices reduce the permeability of the material by filling the pores [24]. However, as long as their mass percentage remains moderate, the water continues to circulate in the interstices without much problem. On the other hand, from a certain value (related to the sieving curve of the material), the fines favour the accumulation of the water in the material rather than the circulation of the latter. Thus, as already mentioned by Casagrande [26], the criterion concerning the content of elements with a diameter of less than 20 μm should be less than 2%. Currently, on the basis of the different experiments carried out since then and for practical reasons (the mechanical sieving stops at 63 μm), the majority of the specifications limit the content of fines (Ø < 63 or 80 μm) to a lower or equal value to 7% for layers with draining role, such as the sub-foundation.

(c) Frost sensitivity: in case of frost, stationary water freezes more easily than moving water. The transformation from liquid to solid state causes an increase in the volume (swelling). This swelling is characterized either by an uplift of the upper structure or by an increase of stresses on the different materials [27]. This therefore facilitates the breakage and the production of fines and accelerates the phenomena of mechanical degradation of the material. A non-draining and therefore rich in fines structure accelerates the propagation of frost and thaw fronts.

These considerations are of particular concern when using recycled concrete aggregates (RCA) which are more and more used in road foundations [27,28]. RCA are composed of a mix of natural aggregates and adherent hardened cement paste [29,30,31,32]. The latter is usually much more porous than natural aggregates [33,34] and leads to a large water demand which makes RCA less easy to recycle into concrete [1,35,36,37]. Properties of RCA such as water absorption and porosity can deeply influence the fresh properties of concrete as well as mechanical properties and durability of concrete made with RCA [16,38,39,40,41]. Over the past decades, many researchers have investigated the possibility of using C&DW materials in the road base and sub-base [27,42,43,44,45,46,47]. They confirm that the properties of recycled aggregates could influence the performances of unbound road layers [27,43,48,49,50].

Molenaar and van Niekerk [45] investigated the effects of gradation, composition and degree of compaction on the mechanical characteristics. The results showed that crushed recycled concrete and masonry rubble can be used to produce good quality road bases. The results indicated that the composition, particle size and degree of compaction have a strong influence on the mechanical properties of recycled materials, but the degree of compaction is the most important influencing factor. This was an important conclusion for construction in practice, since the degree of compaction is much easier to realize and to control than the other parameters.

Park [46] investigated the characteristics of RCA as base and sub-base materials. The results showed that RCA can be used as alternative materials to crushed natural aggregates for roadways. The stability and shear resistance of RCA in wet conditions were lower than in dry conditions, and the reduction rate was comparable with that of natural aggregates. In addition, the deflection of the RCA section was similar to that of natural aggregates section in the field.

Poon and Chan [47] presented the feasible use of RCA and crushed clay brick as unbound road sub-base materials. The results demonstrated that the use of 100% of RCA decreased the maximum dry density and increased the optimum moisture content of the sub-base materials compared to those of natural sub-base materials. The California bearing ratio (CBR) value of sub-base using crushed clay brick was lower than that with RCA. Nevertheless, the soaked CBR value for all recycled sub-bases were 30% greater than that of the minimum strength requirement in Hong Kong.

Leite et al. [44] evaluated the composition and compaction influences on the mechanical behaviour of road base and sub-base layers based on the C&DW materials. The CBR value and the resilient moduli obtained with recycled C&DW materials were similar to those obtained with natural aggregate commonly used in road construction.

Barbudo et al. [42] studied the relationship between different constituents of recycled aggregates and their mechanical behaviour for the road application through a statistical analysis. The results showed that the correct selection of materials and the removal of impurities in a plant with pre-screening and double crushing are important to improve the quality of recycled aggregates. They concluded that recycled aggregate can be used as sub-base materials in roadways.

Jiménez et al. [51] evaluated the behaviour and environmental impact of recycled aggregates from selected C&DW in field conditions. The results demonstrated that both recycled aggregates (4/40 mm, recycled concrete aggregate and recycled mix aggregate) were of good quality and met all limits. The static plate load test showed an excellent bearing capacity in both structural layers. In addition, the use of recycled aggregates in unpaved rural roads did not present a risk of environmental contamination to ground and underground water.

Arulrajah et al. [11] investigated the possibility of using RCA, crushed brick (CB), reclaimed asphalt pavement (RAP), waste excavation rock (WR) and recycled glass in unbound pavement base/sub-base applications. RCA, CB and WR were found to meet the physical and shear strength requirements for aggregates in pavement base/sub-base applications. RAP and recycled glass have to be blended with higher quality aggregates to further enhance their physical and strength properties, particularly the Los Angeles abrasion and California bearing ratio in order to meet road authority requirements.

Sangiorgi et al. [52] focused on the development of the stiffness of recycled materials during construction, as well as how it modified over time. An experimental embankment with four sections of different recycled materials was tested and fields were made from two structural layers, forming a homogenous thickness of about 80 cm. The structural performance of the embankment was determined using different types of lightweight deflectometers. The results showed that recycled aggregates performed well when properly compacted and may showed some positive self-cementing properties.

Silva et al. [53] presented the representative case studies of several applications undertaken in several countries worldwide, namely recycled aggregates in unbound in road and building constructions. For some time now, there have been several real-scale road applications comprised of recycled aggregates, the experience of which has indicated the material’s technical and economic viability.

Finally, some authors investigated the permeability of mixtures prepared with varying RCA contents [13]. The results showed that the use of 75% RCA allowed permeability values closer to those obtained in natural aggregates. However, the influence of fine particles into recycled aggregates on the performance of road foundation is still limited [27,43,54], in particular the shape and grading during the construction procedure and weathering ageing have not been previously reported [34,43].

This study focuses on the recycled fine C&DW specific characterization for exploring the effect of recycled fine materials in road construction. The main objectives of this study are twofold: firstly, the influence of treatment in recycling plants on the properties of recycled aggregates has been explored. Afterwards, an experimental road foundation has been built and material has been excavated for analysing the evolution of particles and materials induced by the construction process and environmental ageing. In addition, special attention has been provided for the shape analysis of fines.

## 2. Materials

Recycled aggregates have been collected from FEREDECO, Fernelmont, Belgian recycling plants for analysis and characterization [55]. Four types of samples were investigated, specifically fine particles.

(1) Raw materials (M84). Four raw materials (noted as M84A, M84B, M84C and M84D, respectively) were directly collected from four different recycling sites. The quantity of each raw material was about 20 m^3^ and the origin of the raw material was unknown. A pre-treatment operation was necessary before sampling. This operation consisted of eliminating the fraction greater than 100 mm by means of a comb screen mounted on a mobile unit of treatment. In order to facilitate manual sorting on the different raw samples, a sieving separation was first performed on each of them. The sample was fed on a conveyor belt equipped with a magnetic pulley at its end which removed the ferrous metal elements from the flow of material [56]. Then, a double pass was provided on a double-stage vibrating screen in order to obtain four particle size fractions in order to carry out the visual characterization: fraction (+40 mm); fraction (−40 + 16 mm); fraction (−16 + 4 mm); and fraction (−4 mm).

Four samples referenced M84Af to Df (these represent the fraction under 4 mm) were collected from materials M84A to D, respectively.

(2) Recycled materials after treatment (M37). Four recycled materials (noted as M37A, M37B, M37C and M37D, respectively) were produced by means of a crushing treatment of M84 samples (M84A, M84B, M84C and M84D, respectively) after decontamination [56]. The main steps of a C&DW recycling facility are as follows [57]:

(a) Scalping: pre-screening in order to separate fine particles and soil before crushing;
(b) Crushing (primary and eventually secondary);
(c) Iron extraction by electromagnetic strip;
(d) Manual extraction of impurities;
(e) Screening to the desired fraction.

Four samples referenced M37Af to Df (these represent the fraction under 4 mm) were collected from materials M37A to D, respectively.

(3) Recycled materials after compaction (CRR11). Experimental road foundation (10 × 3 × 0.3 m) was built with M37A (0/31.5 mm) and material was excavated after one week for analysing the evolution of particles and materials induced by the construction process. The treated recycled material M37A was spread by means of a blade over the entire surface of the board (over a thickness of around 30 cm) with the incorporation of temperature sensors at different depths. The compaction of the layer using a BOMAG BW 213D compactor (Bomag, Hamme, Belgium) according to the method commonly used on site by the contractor, namely four passes with a low amplitude vibrating roller and two passes with a non-vibrating roller in order to close the layer. CRR11f refers to the fraction lower than 4 mm from CRR11.

(4) Recycled materials after freeze–thaw cycles (CRR22). These materials were the same as CRR11 but were exposed outside without cover after 6 months’ natural environmental ageing and 17 recorded freeze–thaw cycles (December 2014–May 2015, Belgian climate conditions). The lowest recorded freeze and highest thaw temperature were −4 °C and 12 °C, respectively, according to the sensors on the layer’s surface. CRR22f refers to the CRR22 fraction under 4 mm.

## 3. Experimental Methods

Bags of all studied materials (described in the section of materials) were collected (about 1 ton for each sample) and representative samples (about 30 kg for each sample) were obtained by coning and quartering. The physical characteristics including the density, the water content, the capacity of water capture from atmospheric air as well as the particle size distribution were conducted on the representative samples. The mineralogical and chemical compositions of the studied materials were investigated by X-ray diffraction (XRD) and X-ray fluorescence (XRF), respectively. In addition, the chloride and sulphate contents were investigated on these fines materials. The mechanical characterization was carried out by means of Los Angeles (LA) abrasion, micro-Deval resistance and static plate load tests. The shape of the studied materials was also performed by means of an image analyser. Table 1 presents the summary of the experimental program conducted in this study. The detailed information has been given in the following sections.

### 3.1. Physical Characterization

The constituents of the raw materials M84 were measured according to the European standard EN 933-11 [58]. Each coarse fraction (larger than 40, 16/40 and 4/16 mm) of raw materials (M84A to M84D) was evaluated on the representative samples after pre-drying in the oven of 60 °C (about 3 kg). The constituents of the total fraction of coarse aggregates (larger than 4 mm) were estimated on the basis of the constituents and fraction distribution of each fraction.

The particle size distribution of the studied materials was determined using the European standard EN 933-1 [59]. For determining grain size distribution and in order to avoid the loss of possible soluble elements, the “dry” technique was selected, with the mesh sieves of 4, 2, 1, 0.5, 0.25, 0.125 and 0.063 mm, respectively, superimposed on a vibrating table for 15 min (60 and 80 vibrations/s).

The representative samples were pre-dried in the oven at a temperature of 105 °C. The bulk density ($\rho_{b}$) was estimated by filling a graduated cylinder with a determined volume of dried samples (*V*). The mass (*m*) of this known volume of matter allowed to calculate its bulk density (Equation (1)). The absolute density of specimens (the mass of a particle divided by its volume, excluding open and closed pores) was measured using helium pycnometer:

(1) $$\rho_{b}=m/V$$

**Figure 1:**
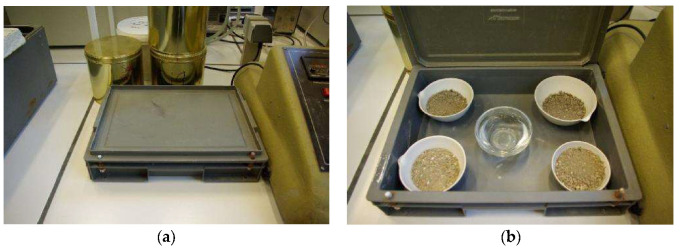
Experimental device for water capture measurement from atmospheric air water: (**a**) closed box in the laboratory with temperature and humidity set at 20 ± 3 °C and 60 ± 10%; and (**b**) the closed box containing the dried samples and water.

In order to measure the water content of the raw materials, the samples were stored in the laboratory at a constant temperature and hygrometry (21 °C and 60% relative humidity) for at least 1 month (as close as possible to the “natural” conditions). The water content of samples reaching constant state at laboratory condition was determined using the European standard EN 1097-5 [60]. After the drying operation in the oven at a temperature of 105 °C, the representative samples were placed in a closed box containing water (Figure 1) in order to determine their capacity of water capture from atmospheric air (the external temperature and humidity were set at 20 ± 3 °C and 60 ± 10%). This original test is very important with regard to the behaviour of road foundation in case of freezing. Moreover, higher water capture is related to a higher amount of fine particles. The mass variation of the samples was regularly registered.

### 3.2. Mineralogical and Chemical Characterization

The mineralogical characterization was performed by means of X-ray diffraction (XRD). The samples were dried and milled before being subjected to X-ray diffractometers (PHILIPS PW-1130/90, Philips, Amsterdam, Netherlands). The position and the relative intensity of the powder diffractogram peaks made it possible to determine the nature of the constituents. The semi-quantitative analysis with X-ray fluorescence spectrometry (spectrometer ARL 9400 Sequential XRF XP, ThermoFisher, Waltham, MA, USA) was determined according to the protocol described by [61]: the accuracy of the results is of the order of 5% (presented as an indication). The Losses On Ignition (LOI) at 500 and 1000 °C, the chloride content of aggregate and the sulphate content (acid soluble) of aggregate were measured using the European standard EN 1744-1 [62].

### 3.3. Mechanical Characterization

The resistance to wear of samples was determined by means of micro-Deval coefficient which corresponds to the percentage of original sample reduced to a size smaller than 1.6 mm during rolling [63]. The fraction of samples (10/14 mm) was tested with the addition of water. The drum within samples (pre-dried in the oven at 105 °C, noted as *M_a_*) and ball load (5000 ± 5 g) were rotated at a speed of 100 ± 5 rounds per minute for 12,000 ± 10 revolutions. After the rotation, the materials were collected and washed carefully on the 1.6-mm sieve, and then dried in the oven at 105 °C until constant mass (noted as *M_b_*). The micro-Deval coefficient (*M_DE_*) is calculated according to EN 1097-1 [63] as follows (Equation (2)):

(2) $$M_{DE}\left(\%\right)=\left(M_{a}-M_{b}\right)/M_{a}\times 100$$

The Los Angeles (LA) abrasion test of samples was measured according to the European standard EN 1097-2 [64]. The LA abrasion test was carried out using aggregate sizes (10/14 mm fraction). Aggregates (5000 g) were dried at 105 °C for 24 h and then cooled to room temperature before placing in a steel drum. The drum was rotating for 500 revolutions at a rate of 31–33 rev/min, along with nine steel spheres (approximately 4840 g). After the 500 revolutions were complete, the crushed aggregate particles were sieved through a 1.6 mm sieve. The amount of material passing the sieve, expressed as a percentage of the original mass, is the LA abrasion resistance.

During the road foundation construction built with M37A (0/31.5 mm), the dry density and water content of the layer were determined using a gamma densimeter at two different locations; this system is widely used in France and Belgium for road construction control [65]. The results were compared with the maximum dry density and optimum moisture obtained with the Modified Proctor Test [66] to verify that the materials had been correctly set. To measure the bearing capacity of the layer, static plate load tests were conducted in accordance with CME 50.01 [67]. The results of the plate load test are presented as curves of applied contact pressure versus settlement. The bearing capacity *M_i_* (*M*_1_: 1st plate loading; *M*_2_: 2nd plate loading) is determined by the Equation (3). A 309-mm diameter steel bearing plate was used for this test. The compressibility coefficient (m) is determined by the ratio of *M*_2_/*M*_1_.

(3) $$M_{i}=D\times \left(\Delta_{p}/\Delta_{s}\right)$$

where *D* is the diameter of the plate (in cm); Δ_*p*_ is the difference of applied contact pressure (in MPa); Δ_*s*_ is the difference of the settlement (in cm).

### 3.4. Shape Analysis

**Figure 2:**
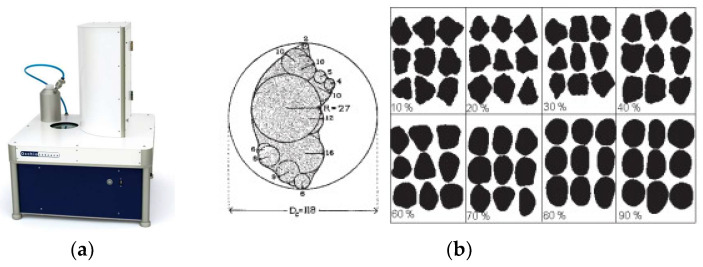
Occhio 500 Nano image analyser (**a**) and Occhio Bluntness values (**b**) [71].

Static image analysis was performed by means of the Occhio 500 Nano image analyser (Figure 2, Occhio, Liège, Belgium) and a high-resolution scanner EPSON V750PRO (Table 2, Epson, Tokyo, Japan) [68]. This instrument includes an integrated vacuum dispersion system and a high-quality optical component which allows assessing size and shape of a set of dispersed particles. Few milligrams of particles were dispersed onto a circular glass slide which is moved (in front of) above a collimated blue (490 nm) LED backlighting. Pictures of individual particles were captured with a 1392 × 1040-pixel video camera fitted with a telecentric lens. The system routinely determined the magnification through the imaging of a calibrated grid. During this experiment, the working resolution was set up to 0.563 µm/pixel. The inscribed disk diameter of each particle was calculated in real time to build particle size distribution curves weighted by apparent volume (PSD-V’) [69], making the assumption that particles have identical densities and flatness ratios, whatever their size.

Particle image acquisition method proceeded by scanning the first 50,000 particles, ensuring that particles are scanned at least once on the whole diameter of the glass slice. The accuracy associated to the estimation of Particle Size Distribution PSD-V’, expressed as the two-sided 95% confidence interval, was computed by means of the bootstrap method [69,70]. Regarding the large number of particle analysed, any further filtering was required to obtain reliable results.

Raw materials (M84), recycled materials after treatment (M37) and recycled materials after compaction (CRR11) were sieved and divided into eight different grades (Table 2).

The selected discriminant parameters for shape analysis were [71]:

(1) Elongation (from inertia ellipse)
  1−Ell.Width/Ell.Length
  where *Ell.Width* and *Ell.Length* are the width and length of the particle on the inertia ellipse minor and major axes, respectively. Elongation with default value of 1 if *Ell.Length* = 0.
(2) Convexity
  Perimeter/Convex Perimeter
  where *Perimeter* is the perimeter length calculated by multiplying 1 for each link between horizontal and vertical pixels and √2 for other links. *Convex Perimeter* is the sum of the lengths of the convex perimeter segments. If the convex perimeter contains only three segments or less, *Convex Perimeter* = 1.
(3) Occhio Bluntness

The calculation of the Occhio Bluntness was based on Eric Pirard’s thesis; this parameter is related to the variability of the particle calliper distribution [71]. The calliper is the distribution of rays of circles tangent to the outer pixels and included in the particle. The bluntness values are shown in Figure 2.

## 4. Results and Discussions

### 4.1. Physical Characterization

Mass distributions of each fraction of raw material (M84) are shown in Table 3. Mass distributions were calculated without considering the metal fraction (2 kg per ton). On the basis of visual observations, it can be concluded that all the raw materials are composed of many coarse elements such as concrete blocks and bricks; however, the raw materials M84A to M84C present a brown colour, but a grey colour is presented for M84D. The raw material M84C presents the highest content of fine material (fraction smaller than 4 mm), which is due to the origin of C&DW. Table 3 shows the classification of the components of different materials. The raw material M84B offers the highest clay masonry content (e.g., bricks and tiles, Rb = 57.1%) and the lowest content of concrete and unbound aggregate (Rc + Ru = 37.9%). On the contrary, the raw material 84C has the lowest clay masonry content (Rb = 22.2%) and the highest content of concrete and unbound aggregate (Rc + Ru = 72.0%), which is within the requirement for type B recycled aggregates according to European standard EN 12620 [72].

After the treatment in the recycling plant, the clay masonry content of all four recycled materials M37 decreased and all of them were finally within the requirement for type B recycled aggregates. The recycled materials after treatment M37A has the lowest clay masonry content (Rb = 8.9%) and the highest content of concrete and unbound aggregate (Rc + Ru = 88.0%). The recycling process including pre-screen, crushing, sorting and decontamination (ferrous element, wood, plastic and paper) in four C&DW recycling facilities can decrease the quantity of bricks and tiles present in the coarse fraction 4/30 mm. The quantity of fraction 0/4 mm, however, increases after the treatment (from 29.5% to 33.7% for M84A; from 21.9% to 26.5% for M84B; from 28.6% to 36.5% for M84D) except for M37C, which means that the crushing process will produce larger quantity of sand and filler fractions.

**Figure 3:**
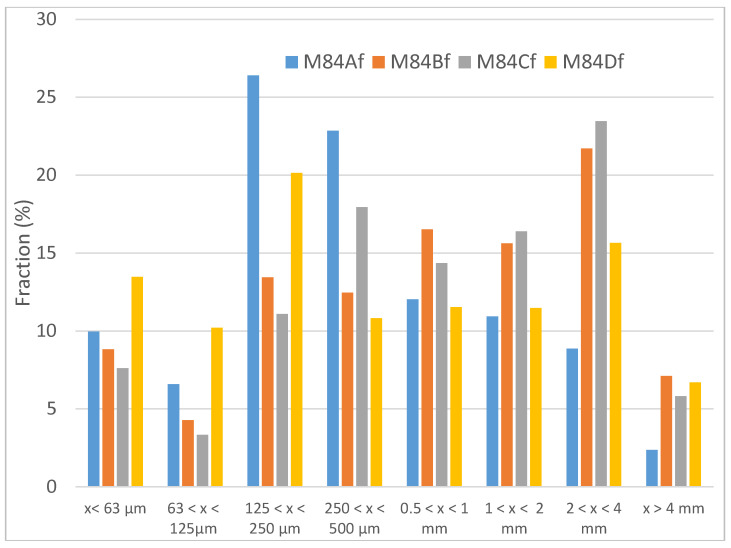
Percentage of fractions obtained for the raw materials samples (M84Af to Df).

On the basis of visual observations, M84Bf to Df samples are dark brown while M84Af is light brown. It is important to underline that, in view of the presence of condensed water on the bag in which the samples were stored, all these powders seem to have an important absorption or adsorption capacity. Figure 3 presents the distribution of the different fractions obtained during particle size separation for each sample. Regarding the distribution of particle size fractions in the different samples, M84Af and Df contain the highest content of fine particles (<250 μm). However, M84Df has the highest concentration of particles with the fraction (0 to 125 μm). M84Af is essentially formed of fragments whose particle size is between 125 and 500 µm. It is also in this sample that we find the least coarse elements (>2 mm). From a sieving point of view, M84Bf and M84Cf are very similar. These two samples are also containing the coarsest elements, essentially between 500 μm and 4 mm, and their cumulative refusal percentages are systematically higher than those of the other two curves. The difference between the particle sizes of these two samples is marked at the level of 250–500 μm, which is enriched in the fine fraction for M84Bf.

**Figure 4:**
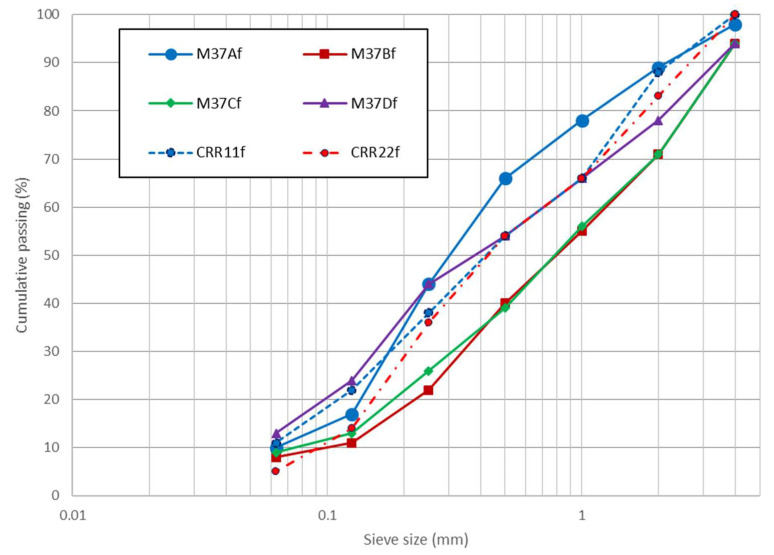
Particle size distribution of the sable fractions of the studied samples.

Figure 4 shows the sieving curves of recycled materials after treatment, compaction and freeze–thaw cycles. After compaction process, the fine particle percentage (fraction 0/125 µm) slightly increased according (curve CRR11f), which means that some breaking occurred during compaction. The materials seem however to remain reasonably well graded through the compaction process. This is consistent with the results of previous studies [30,44]. After the freeze–thaw cycles, the sieving curve of CRR22f sample did not specifically reveal the formation of fines in larger amounts, which means that the freeze–thaw cycles do not affect the particle size distribution for these recycled materials. During the freeze–thaw cycles, the risk of damage could be due to the penetration of water into the particle pores upon freezing, thus creating considerable tensile stresses that can break aggregate particle [43]. These recycled aggregates showed good resistance to the freeze–thaw cycles.

Table 4 presents the water content of the raw materials (M84Af to Df) after reaching the constant state in the laboratory condition (21 °C and 60% relative humidity). The water content of the sand fraction of raw materials ranged from 11.9% to 14.7%. It revealed that the presence of fine particles is able to physically attract water: the finer the particles, the higher water content. This is specifically the case for M84Df, which has the highest content of fine particles under 63 microns.

M84Df had the highest bulk density comparing with other three raw materials (the density of M84Df was 919 kg/m^3^, while it was 1042 kg/m^3^ for M84Bf in Table 4). It is difficult to draw conclusions from these values without performing other types of measurements. Indeed, as previously stated, two parameters (intrinsic density of the materials and packing density) intervene in this density, making the interpretation difficult. However, the materials containing high water content and the finest particles content offered the lowest bulk density (M84Df).

**Figure 5:**
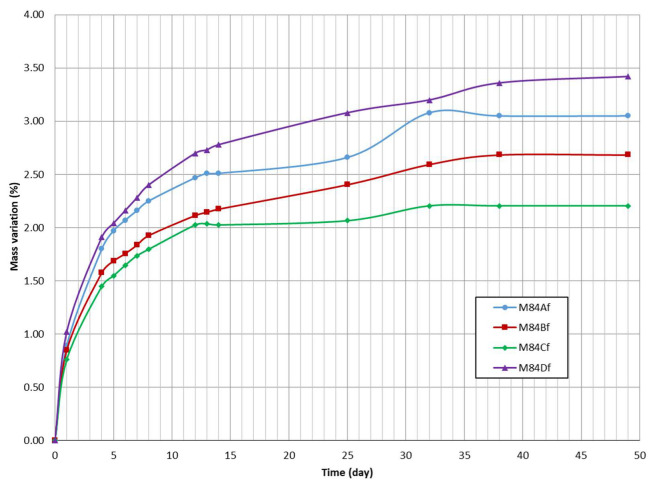
Capacity of water capture from atmospheric air of samples (M84Af to Df).

The mass variation of the samples at laboratory condition is shown in Figure 5. The result showed a larger increase in mass over time for sample M84Df than for sample M84Cf. M84Df sample contains higher amount of small particles, which can induce the creation of pores and favour capillary water absorption. During the first week, the mass increase of the powders was the most consequent. This parameter still increased in the following weeks, but, less importantly, the mass of the different samples continues to progress but much more slowly. It is noteworthy that the results obtained are not correlated with the previously calculated water content. In fact, the M84Bf sample, which has the highest value in terms of humidity, has a lower mass gain vs the two other samples. Even more surprising, M84Af, which has a relatively large mass gain, offers the lowest water content.

Physical properties of recycled materials after treatment (M37f) were broadly similar to those of raw samples (M84f). However, higher absolute density of specimen collected after compaction (CRR11f) and freeze–thaw cycles (CRR22f) could be correlated to higher fine content, even if the difference maybe not discriminant.

### 4.2. Mineralogical and Chemical and Characterization

Table 5 and Table 6 show the mineralogical and chemical compositions of the materials, respectively. Powder diffractograms did not show significant differences between the four samples of raw materials. They are, in fact, essentially composed of quartz and, in smaller proportions, of calcite, plagioclase and potassium feldspars: cementitious components could also be present in the material and interfere with interpretation. However, the presence of the minerals belonging to the group of clays in the M84Bf to Df samples were observed, whereas M84Af did not seem to contain them. However, we must pay attention to this last statement: the absence of peaks in clays in the latter may be due to the absence of these compounds or their presence in too low levels to be detected. It indicated, incidentally, that no risk of swelling clay effect had to be suspected.

After treatment in a recycling plant, the powder diffractograms (Table 5) did not show significant differences between samples, except for M37Af which contains many MgO-based compounds. Fine materials are essentially composed of quartz (a little more for the sample taken after freeze–thaw) and, in smaller proportions, of calcite, plagioclase and potassium feldspars. The high rate of potassium feldspars revealed the presence of large quantities of clay materials, specifically for CRR11f where possible contamination during sampling is envisaged. Finally, X-ray fluorescence spectrometry allowed an evaluation of the proportion of the different compounds present in the samples (Table 6) and confirmed a majority of SiO_2_ and CaO, which probably means a discriminant part of cementitious original products. The compaction process and freeze–thaw cycles seem to have no effect on the mineral compositions of materials.

Loss on ignition (LOI) at 500 °C decreased for all raw materials after the treatment (Table 7). The recycling process including pre-screen, crushing, sorting and decontamination (ferrous element, wood, plastic and paper) in the four C&DW recycling facilities could improve the quality of recycled materials by reducing the organic content. There was little difference between the samples, specifically at 1000 °C where it is possible to probably detect decomposition of calcite and magnesium carbonates [73].

Chloride content of all studied materials is at a low level (Table 7). The chloride may often come from de-icing salts sprayed during winter on civil engineering structures [36], even if in very small quantities in the present specimens. After the treatment and compaction process the chlorides content remain low and do not seem to be a risk for their use in cementitious mixtures. On the other hand, the risk of contact with steel elements is unlikely at the level of base/sub-base road applications in which these recycled materials are used.

The acid-soluble sulphate content values obtained for four raw materials decreased significantly after the treatment. The origin of sulphates can be attributed to gypsum and plaster used in buildings. After the treatment, all four samples were below the limits (0.8%) imposed according to Del Rey et al. [48]. It indicated that the decontamination of gypsum-based construction materials is efficient for all the four recycling facilities. The solubility of sulphates should be restricted in road materials to guarantee the dimensional stability of the section and to avoid potential adverse effect (expansive disruption) due to the presence of sulphate near or in contact with concrete structure [74].

The investigations on raw fine materials do not reveal major differences, whatever the recycling plant and the possible origin—building or civil engineering structures—of the materials: mostly containing SiO_2_, with very low values of lime, sulphate, chlorides and inorganic materials, they mainly vary from sieving curve and sieving grades. The investigations on fine materials after the treatment showed that the quality of recycled aggregates (including the sulphate content, organic content) were improved. The removal of impurities (such as organic matters, plastic, gypsum-base construction materials) and a pre-screening at the beginning of the recycling process are very important, specifically for the quality and the quantity of fine particles. Thus, the recycled aggregates from C&DW after treatment and good control can be used as sub-base materials in road construction. In addition, the compaction process and freeze–thaw cycles seem to have no or little effect on the mineral and chemical properties of the studied materials.

### 4.3. Mechanical Characterization

Table 8 shows the resistance to wear of samples determined by the micro-Deval coefficient and LA abrasion resistance. An LA abrasion maximum value of 40 is normally adopted by road authority specifications for road foundation construction [75]. The M37B and CRR11 sample meets these maximum criteria, while the other samples are slightly above the limits. The micro-Deval coefficient maximum values of 35 and 50 are required for primary roads (type I and II_a_) and secondary roads (type II_b_ and III), respectively, according to road authority specifications for road foundation construction [75]. Three of the four samples meet the maximum criteria for secondary roads foundation construction. This suggests that the recycled aggregates from C&DW may have to be blended with other aggregates to enable its usage in specific road foundation (for example for primary roads). After the compaction, the LA abrasion resistance and micro-Deval value are slightly decreased, which means that the wear resistance of recycled aggregates was slightly affected by the construction process. In addition, the freeze–thaw cycles seem to have no or little effect on the wear resistance of studied materials.

The optimum moisture content of M37A is obtained as 12% and the maximum dry density is determined as 1.85 g/cm^3^ according to the laboratory Modified Proctor test. The results of gamma densimeter (Table 9) show that the compaction degree varies from 83.13 to 90.06% of the optimum laboratory Modified Proctor test condition. The compaction is therefore insufficient with regard to the specifications for road foundation (95%). However, the compaction water content (Table 9) is lower than the optimum moisture content obtained in laboratory (12%), which is possibly due to the lower compaction degree on site than that of the laboratory condition. The compaction degree increases after 17 freeze–thaw cycles (six months after the construction). The higher water content values after the freeze–thaw cycles are obtained, which is possibly due to the rainwater penetration several days before test. To measure the bearing capacity of the layer, static plate load tests has been carried out after the compaction. A bearing capacity M_1_ of 33.2 MPa was obtained (Table 10), which is slightly lower than the specifications for the road foundation (35 MPa) [75]. The compressibility coefficient m is relatively high (>2.5, Table 10). The bearing capacity (M_1_) after freeze–thaw cycles is twice higher than that after its compaction. This could be due to self-cementing properties of fine fraction of recycled aggregates (unhydrated cement and C_2_S present in the adherent mortar of fine RCA, especially the active fractions of 0–0.15 and 0.3–0.6 mm as reported by Poon et al. [76] and Sangiorgi et al. [52]). Poon et al. [76] indicated that the grade, age and amount of cementitious materials used in the original concrete would be the deciding factors on the self-cementing properties of sub-base materials. Furthermore, the fine particle percentage (fraction 0/0.125 mm) slightly increased and some breaking occurred during compaction: the grading of RCA has thus changed after compaction process. The effects of the compaction effort and the rate of the compaction could favour the self-cementing properties of sub-base materials. In addition, the drainage of the sub-base materials could also improve the bearing capacity of road foundation.

### 4.4. Shape Characterization

The results of shape analysis are presented from the coarsest to the finest particle size fraction, in graphical form. The legend is shown here below:

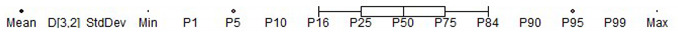



**Figure A1:**
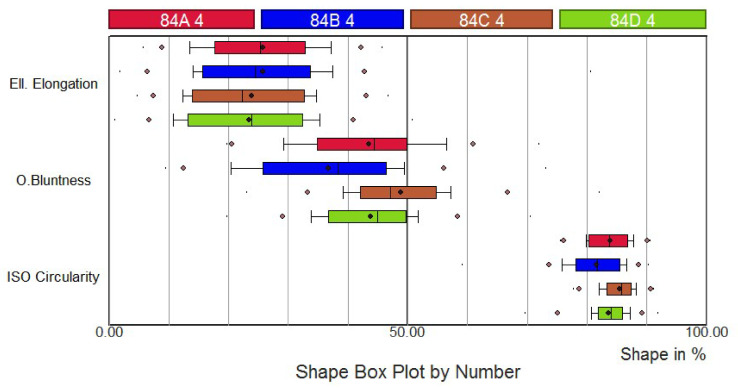
Shape analysis of particles (>4 mm) (84A, 84B, 84C and 84D refer to raw materials M84Af, M84Bf, M84Cf and M84Df, respectively).

The samples (84Af, 84Bf, 84Cf and 84Df) contained small quantity of particles with a particle size greater than 4 mm, while the samples (37Af, 37Bf, 37Cf, 37Df and CRR11f) contained only the fraction lower than 4 mm (the shape analysis of particles larger than 4mm is shown in Figure A1 in Appendix A). It should be noted that these analyses focused on only 63 to 130 particles, which is quite low for a precise shape characterization. No definitive conclusion can therefore be inferred as regards this particle size fraction.

**Figure 6:**
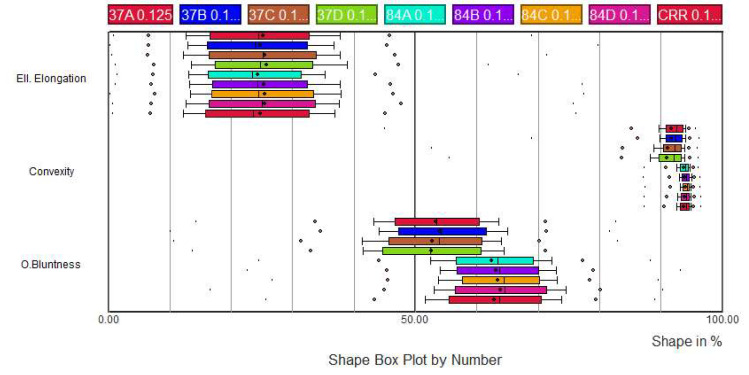
Shape analysis of particles (0.125–0.25 mm) (84 refers to raw materials: 84A, 84B, 84C and 84D refer to M84Af, M84Bf, M84Cf and M84Df, respectively; 37 refers to recycled materials after treatment: 37A, 37B, 37C and 37D refer to recycled materials after treatment M37Af, M37Bf, M37Cf and M37Df, respectively; CRR refers to recycled materials after compaction: CRR11f).

**Figure 7:**
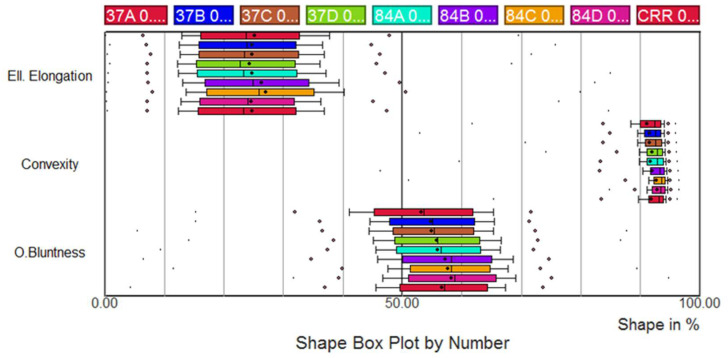
Shape analysis of particles (0.063–0.125 mm) (84 refers to raw materials: 84A, 84B, 84C and 84D refer to M84Af, M84Bf, M84Cf and M84Df, respectively; 37 refers to recycled materials after treatment: 37A, 37B, 37C and 37D refer to recycled materials after treatment M37Af, M37Bf, M37Cf and M37Df, respectively; CRR refers to recycled materials after compaction: CRR11f).

**Figure A2:**
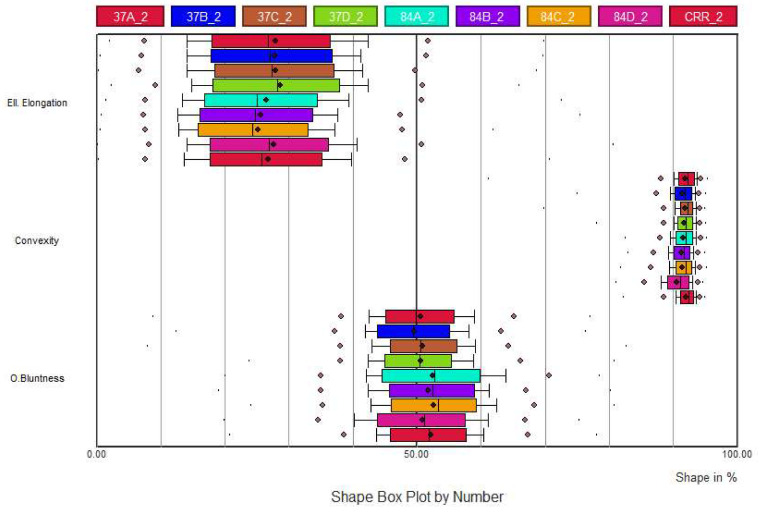
Shape analysis of particles (2–4 mm) (84 refers to raw materials: 84A, 84B, 84C and 84D refer to M84Af, M84Bf, M84Cf and M84Df, respectively; 37 refers to recycled materials after treatment: 37A, 37B, 37C and 37D refer to recycled materials after treatment M37Af, M37Bf, M37Cf and M37Df, respectively; CRR refers to recycled materials after compaction: CRR11f).

**Figure A3:**
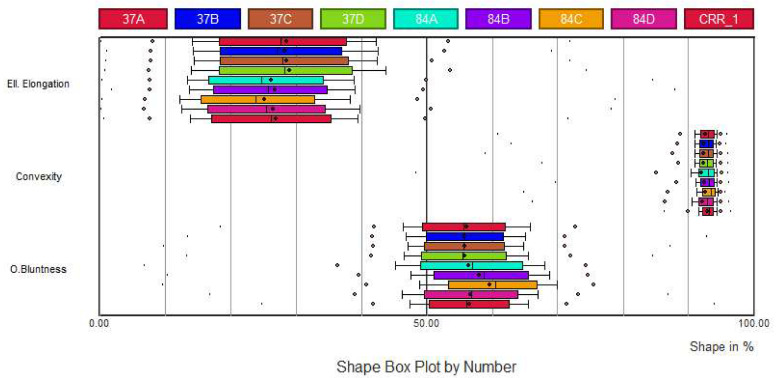
Shape analysis of particles (1–2 mm).

**Figure A4:**
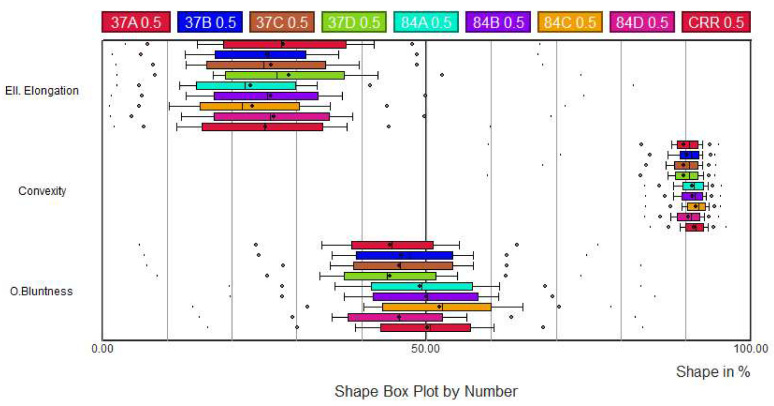
Shape analysis of particles (0.5–1 mm).

**Figure A5:**
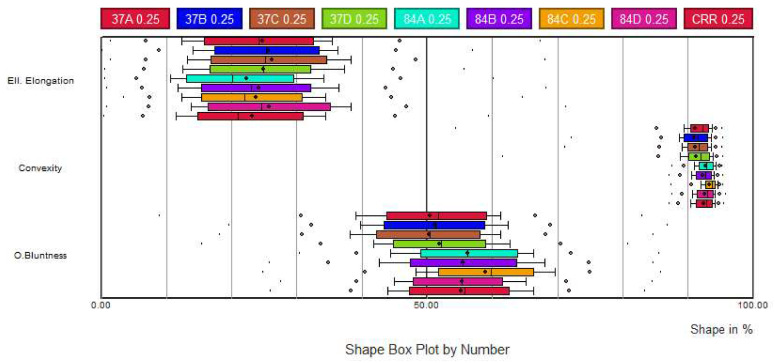
Shape analysis of particles (0.25–0.5 mm).

**Figure A6:**
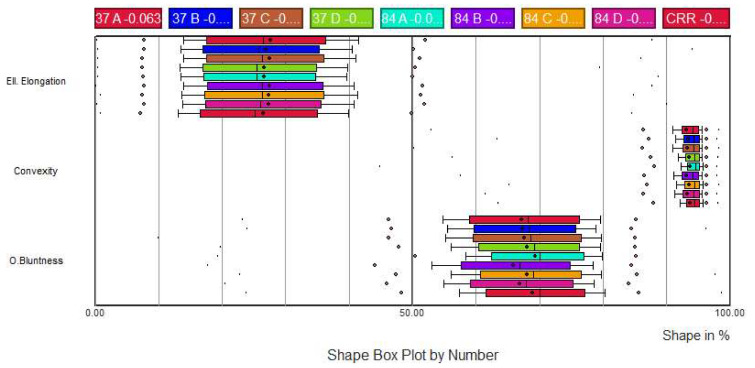
Shape analysis of particles (0.020–0.063 mm).

Between 500 and 4000 particles have been analysed, depending on the particle size: the larger the size, the lower the number of analyses (Figure 6 and Figure 7 and Figure A2, Figure A3, Figure A4, Figure A5 and Figure A6 in Appendix A).

An image treatment was applied on granular fraction 0.020–0.063 mm for removing particles smaller than 0.020 mm (Figure A6 in Appendix A). Indeed, below this dimension, the number of pixels used in the shape measurement is too small to obtain significant results. About 8000 particles were analysed for each sample.

Significant differences in shape were observed for the nine samples in each of the granular fractions tested. Most of the time, the trends were consistent for the same sample from one granular fraction to another. The raw sample M84Cf contained globally more rounded particles than all the others, in particular because of the coarse particles. However, for fines (<0.125 mm), raw sample M84Df had significantly higher bluntness values. In the size fraction 0.125–0.25 mm, and to a lesser extent for the other fractions, the difference between the raw samples M84 and the recycled samples after treatment M37 was particularly visible. Raw fine materials (M84) globally offered higher bluntness but equal elongation and convexity than recycled fine materials (M37). The recycling process, particularly the crushing process, could modify the particle shape [30]. No major differences between the four recycled samples (M37A to D) appeared. After the compaction process, the values of Occhio bluntness and convexity increased slightly, while the elongation values remained, despite some breakdown that occurred during compaction.

## 5. Conclusions

In this study, the effects of recycling process, compaction and freeze–thaw cycles on the properties of recycled fine aggregates obtained from C&DW used for unbound sub-base were evaluated. In addition, special attention was also provided for the shape analysis of fines. The following conclusions may be reached from the present investigations concerning the analysis of properties of the fine particles (under 4 mm) produced by recycling C&DW:

- The recycling process including pre-screening, crushing, sorting and decontamination (ferrous element, wood, gypsum-based construction materials) could decrease the quantity of bricks and tiles present in the coarse fraction 4/30 mm and slightly increase their quantity within a fine fraction. In particular, the sulphate content could be limited by removing impurities and a pre-screening control at the entrance of raw materials;
- Main discriminant parameter is the sieving curve and the proportion of grades. The mixes containing the highest quantity of fine particles, specifically lower than 63 µm, usually induced a higher water demand and a higher capillary rise. These fine particles represent a detrimental product for long term unbounded foundations in road structures;
- Compaction did not seem to have a major effect on the production of fines (despite some breakdown that occurred during compaction) and on the shape of materials. Mineralogy and chemical characteristics remained globally similar after the compaction, which means no specific degradation of materials with construction process. Freeze–thaw cycles had no measurable effect on the sieving distribution of particles, specifically the production of fines, or the mineralogy and chemical characteristics of materials;
- Bluntness was increasing with finer particles, which means higher surface roughness and higher potential interaction with water. Raw fine materials globally offered higher bluntness but equal elongation and convexity compared to recycled fine materials. After the compaction process, the bluntness and convexity increased slightly, while the elongation values remained similar, despite some breaking occurring during compaction;
- The LA abrasion resistance and micro-Deval coefficient values are slightly affected by the construction process, while freeze–thaw cycles seem to have no or little effect on the wear resistance of the studied materials. The material used for unbound road sub-base could meet the maximum criteria for secondary roads foundation construction on the wear resistance criteria. The recycled aggregates from C&DW without sufficient quality may have to be blended with other aggregates to enable their usage in certain road foundation (for example, for high-quality primary roads). The static plate load tests showed that bearing capacity is slightly lower than the specifications for the road foundation after compaction. After 6 months’ drainage and freeze–thaw cycles, the bearing capacity of sub-base materials is improved due to the self-cementing properties of fine RCA and the drainage of sub-base materials. The long term behaviours of road sub-base using fine RCA in full-scale trails (e.g., after several years’ daily traffic) could be interesting to be investigated in the future.

The most important parameter to be checked before using recycled materials as unbound base/sub-base applications is definitely the content of particles finer than 63 µm that has to be strictly limited. This study has been focused on the experimental analysis the effects of recycled fine aggregates from C&DW for the unbound road sub-base. The theoretical modelling of the analysed parameters (especially the effects of properties of recycled aggregates, the compaction process, and freeze–thaw cycles) on the properties of unbound road sub-base could be interesting for the future study.

New processes for Construction and Demolition Waste treatment have been recently set up: washing a grade lower than 4 mm allows to obtain a very good quality sand (as well as for coarser aggregates which are also washed). These products can be actually used for a concrete skeleton and the production of precast concrete products.

## Figures and Tables

**Table 1:** Summary of the experimental program conducted in this study.

Properties of Samples	Equipment or Technique	Standard or Reference
Bulk density	Balance	–
Absolute density	Helium pycnometer	–
Constituents of recycled materials	–	EN 933-11 [58]
Particle size distribution	Sieve analysis	EN 933-1 [59]
Water content	Balance	EN 1097-5 [60]
Mineralogical composition	XRD	-
Chemical composition	XRF	[61]
LOI at 500 and 1000 °C	–	EN 1744-1 [62]
Chloride content	–	EN 1744-1 [62]
Sulphate content	–	EN 1744-1 [62]
Micro-Deval coefficient	–	EN 1097-1 [63]
Los Angeles abrasion	–	EN 1097-2 [64]
Water content of the layer in situ	Gamma densimeter	[65]
Optimum moisture	Modified Proctor Test	[66]
Bearing capacity of the layer in situ	Static plate load test	CME 50.01 [67]
Shape analysis	Occhio 500 Nano Image analyser and EPSON scanner	[68]

**Table 2:** Performances of the test devices used for shape analysis.

Size Range	Nomenclature	Equipment	Approximate Pixel Size (µm)
>4 mm	4	EPSON scanner	21.2
2–4 mm	2	EPSON scanner	21.2
1–2 mm	1	EPSON scanner	21.2
0.5–1 mm	0.5	Occhio 500 Nano	4.4
0.25–0.5 mm	0.25	Occhio 500 Nano	3.2
0.125–0.25 mm	0.125	Occhio 500 Nano	2.2
0.063–0.125 mm	+0.063	Occhio 500 Nano	1.4
<0.063 mm	−0.063	Occhio 500 Nano	0.9

**Table 3:** Classification of raw materials (M84A to D) and recycled materials after treatment (M37A to D) according to EN 933-11 (Rc, Ru, Rb, Ra, Rg, X and FI refer to percentages of concrete products, unbound aggregate, clay masonry units, bituminous materials, glass, other non-floating materials and volume percentage of floating material in %, respectively).

Name of Materials	Fraction	Mass Percentage	Type of Constituents						
			Rc	Ru	Rb	Ra	Rg	X	FI
M84A	x > 40 mm	44.9	32.3	6.2	56.9	3.1	0.1	1.0	0.4
	16 < x < 40 mm	18.2	47.9	11.0	32.9	2.4	0.9	4.4	0.7
	4 < x < 16 mm	7.4	43.9	32.9	16.3	0.3	0.8	5.3	0.7
	x < 4 mm	29.5	–	–	–	–	–	–	–
	x > 4 mm	70.5	37.6	10.2	45.7	2.6	0.4	3.0	0.5
M84B	x > 40 mm	49.4	30.0	1.5	65.6	1.0	0.3	1.5	0.2
	16 < x < 40 mm	21.4	38.4	3.9	52.1	1.7	1.9	1.7	0.4
	4 <x < 16 mm	7.3	46.3	23.3	22.9	0.3	2.2	4.4	0.5
	x < 4 mm	21.9	–	–	–	–	–	–	–
	x > 4 mm	78.1	33.8	4.1	57.1	1.1	1.0	2.6	0.3
M84C	x > 40 mm	28.1	34.0	23.7	37.9	2.6	0.0	1.4	0.4
	16 < x < 40 mm	23.9	49.5	26.4	16.4	5.3	0.2	2.3	0.2
	4 < x < 16 mm	14.5	36.6	57.1	3.7	0.1	0.3	2.0	0.2
	x < 4 mm	33.5	–	–	–	–	–	–	–
	x > 4 mm	66.5	40.1	31.9	22.2	3.0	0.1	2.4	0.3
M84D	x > 40 mm	48.2	42.3	5.4	50.6	0.8	0.0	0.4	0.5
	16 < x < 40 mm	16.1	44.4	2.6	47.2	1.1	0.5	3.7	0.7
	4 < x < 16 mm	7.1	47.3	21.3	22.8	2.7	0.3	4.4	1.2
	x < 4 mm	28.6	–	–	–	–	–	–	–
	x > 4 mm	71.4	43.3	6.3	46.5	1.0	0.2	2.1	0.6
M37A	4 < x < 30 mm	66.3	43.7	44.3	8.9	1.5	0.0	1.1	0.5
	x < 4 mm	33.7	–	–	–	–	–	–	–
M37B	4 < x < 30 mm	73.5	36.6	33.1	24.2	5.7	0.0	0.1	0.2
	x < 4 mm	26.5	–	–	–	–	–	–	–
M37C	4 < x < 30 mm	73.1	49.2	22.2	25.2	0.9	0.0	2.3	0.1
	x < 4 mm	26.9	–	–	–	–	–	–	–
M37D	4 < x < 30 mm	63.5	45.7	33.3	18.0	0.7	0.0	1.4	0.9
	x < 4 mm	36.5	–	–	–	–	–	–	–
CRR11	4 < x < 30 mm	70.5	41.3	24.6	32.1	1.3	0.1	0.3	0.3
	x < 4 mm	29.5	–	–	–	–	–	–	–

**Table 4:** Physical properties of different samples.

Name of Materials	Bulk Density (kg/m^3^)	Absolute Density (kg/m^3^)	Water Content (%)
M84Af	1018	–	11.9
M84Bf	1042	–	14.7
M84Cf	1021	–	12.9
M84Df	919	–	14.1
M37Af	–	2676	3.8
M37Bf	–	2649	6.1
M37Cf	–	2648	8.8
M37Df	–	2635	6.5
CRR11f	–	2628	8.6
CRR22f	–	2720	14.2

**Table 5:** Mineral composition of the different samples determined by XRD (%).

Name of Materials	Calcite	Quartz	Plagioclases	Potassium Feldspars	Clay	Dolomite
M84Af	12	68	7	13	Not detected	–
M84Bf	11	75	4	10	Detected	–
M84Cf	14	71	7	8	Detected	–
M84Df	16	72	5	7	Detected	–
M37Af	18	55	5	5	–	17
M37Bf	30	59	7	4	–	1
M37Cf	20	59	14	4	–	3
M37Df	25	56	4	9	–	7
CRR11f	20	60	≥3	16	–	Not detected
CRR22f	17	74	3	6	–	Not detected

**Table 6:** Chemical composition of the different samples determined by XRF (%).

Name of Materials	M84Af	M84Bf	M84Cf	M84Df	CRR11f	CRR22f
SiO_2_	70.00	60.59	59.87	60.20	60.62	64.19
TiO_2_	0.32	0.35	0.29	0.35	0.35	0.32
Al_2_O_3_	4.47	6.44	5.19	6.58	6.44	6.43
Fe_2_O_3_	2.31	3.10	3.69	2.93	2.89	2.75
MnO	0.05	0.12	0.26	0.06	0.06	0.06
MgO	0.74	0.85	1.25	0.74	0.97	0.97
CaO	9.71	12.38	13.95	10.08	12.35	11.87
Na_2_O	0.17	0.13	0.14	0.16	0.11	0.14
K_2_O	1.05	1.16	0.97	1.42	1.37	1.28
P_2_O_5_	0.12	0.15	0.15	0.23	0.12	0.11
LOI	9.30	12.13	13.32	14.73	10.81	10.02
Total	98.25	97.39	99.09	97.47	96.09	98.14

**Table 7:** Chemical properties of different samples.

Name of Materials	LOI at 500 °C (%)	LOI at 1000 °C (%)	SO_4_^2−^ Content (%)	Cl^−^ Content (%)
M84Af	5.18	8.96	1.568	0.024
M84Bf	4.5	12.30	1.786	0.024
M84Cf	3.46	13.53	0.618	0.016
M84Df	6.78	14.92	1.450	0.044
M37Af	2.25	17.76	0.617	0.020
M37Bf	2.60	12.72	0.722	0.028
M37Cf	2.12	10.01	0.541	0.020
M37Df	2.71	17.76	0.674	0.028
CRR11f	3.61	10.98	0.525	0.022
CRR22f	2.63	9.88	0.331	0.011

**Table 8:** Micro-Deval resistance and LA abrasion resistance of different samples (%).

Name of Materials	M37A	M37B	M37C	M37D	CRR11	CRR22
M_DE_	48	40	50.5	49	45.5	45.5
LA	42	39	42	46	39	42
M_DE_ + LA	90	79	92.5	95	84.5	87.5

**Table 9:** The results of gamma densimeter measurement of road foundation after the compaction (CRR11) and freeze–thaw cycles (CRR22).

	After Compaction (CRR11)		After Freeze–Thaw Cycles (CRR22)			
Depth from Surface (cm)	−5	−15	−30	−5	−15	−30
Water content (%)	10.45	9.11	8.84	15.91	14.36	13.98
Compaction degree (%)	83.13	88.07	90.06	86.16	91.49	94.46

**Table 10:** The results of plate load test for CRR11 and CRR22.

	Bearing Capacity M_1_ (MPa)	Bearing Capacity M_2_ (MPa)	Report m = M_2_/M_1_
After compaction (CRR11)	33.2	110.4	3.3
After freeze–thaw cycles (CRR22)	68.7	181.8	2.65

## Acronyms

C&DW	Construction and Demolition Waste
CB	Crushed Brick
CBR	California Bearing Ratio
CRR11	Recycled materials after compaction
CRR22	Recycled materials after freeze–thaw cycles
LA	Los Angeles
LOI	Loss On Ignition
M84	Raw materials
M37	Recycled materials after treatment
PSD-V’	Particle Size Distribution curves weighted by apparent Volume
RAP	Reclaimed Asphalt Pavement
RCA	Recycled Concrete Aggregates
XRD	X-ray Diffraction
XRF	X-ray Fluorescence
WR	Waste excavation Rock
